# Long-term effects of a web-based cancer aftercare intervention on moderate physical activity and vegetable consumption among early cancer survivors: a randomized controlled trial

**DOI:** 10.1186/s12966-017-0474-2

**Published:** 2017-02-10

**Authors:** Iris M. Kanera, Roy A. Willems, Catherine A. W. Bolman, Ilse Mesters, Peter Verboon, Lilian Lechner

**Affiliations:** 1 Faculty of Psychology and Educational Sciences, Open University of the Netherlands, P. O. Box 2960, 6401 DL Heerlen, The Netherlands; 2 Department of Epidemiology, Optimizing Patient Care, Care and Public Health Research Institute, Maastricht University, P.O. Box 616, 6200 MD Maastricht, The Netherlands

**Keywords:** Cancer survivorship, Web-based intervention, Physical activity, Vegetable consumption, Long-term effect, Computer tailoring, eHealth

## Abstract

**Background:**

The number of cancer survivors is growing. Negative physical and psychosocial consequences of cancer treatment can occur during survivorship. Following healthy lifestyle recommendations is beneficial to increase quality of life and to reduce the risk of cancer recurrence and comorbidities. To meet individual needs, web-based interventions can supply a large population of cancer survivors with easily accessible and personalized information. Evidence concerning the long-term effects of web-based cancer aftercare interventions on lifestyle outcomes is limited. The present study evaluates the 12-month effects of a fully automated web-based cancer aftercare intervention. We investigated whether the previously determined 6-month effects on moderate physical activity and vegetable intake were maintained over 12 months. Possible moderator effects of using specific intervention modules, gender, age, and education were also explored.

**Method:**

A two-armed randomized controlled trial was conducted using online self-report questionnaires among survivors of various types of cancer (*N* = 462). The intervention group had access to the online intervention for 6 months, and the control group received access after 12-months. Multilevel linear regression analyses (complete cases and intention-to-treat) were conducted to explore 12- month effects.

**Results:**

A significant intervention effect after 12 months was found for moderate physical activity (complete cases: *B* = 128.475, *p* = .010, *d* = .35; intention-to-treat: *B* = 129.473, *p* = .011). Age was the only significant moderator (*p* = .010), with the intervention being effective among participants aged younger than 57 years (*B* = 256.549, *p* = .000, *d* = .59). No significant intervention effect remained for vegetable consumption after 12 months (complete cases: *B* = 5.860, *p* = .121; intention–to-treat: *B* = 5.560, *p* = .132).

**Conclusion:**

The online cancer after care intervention is effective in increasing and maintaining moderate physical activity in the long term among early cancer survivors younger than 57 years. Short-term increases in vegetable consumption were not sustained in the long term. These findings indicate the value and potential of eHealth interventions for cancer survivors. Based on the study results, web-based self-management interventions could be recommended for younger cancer survivors (<57 years of age) as a possible method to increase physical activity.

**Trial registration:**

Dutch Trial Register NTR3375. Registered 29 March 2012.

## Background

Cancer represents a large global health problem, with approximately 14.1 million new cases of cancer in 2012, worldwide [1, 2]. Due to aging and improvements in treatment, the number of cancer survivors is growing. As a consequence of primary cancer treatment, cancer survivors’ quality of life (QoL) can be reduced by physical and psychosocial health problems, such as pain, fatigue, anxiety, depression, and work-related issues [3, 4]. Moreover, survivors are at risk of disease recurrence, and comorbid chronic conditions [5]. In particular, survivors who smoke, are physically inactive, or overweight are at increased risk for mortality, morbidity, and disability [6–8]. A healthy dietary pattern has been associated with a lower risk of obesity, hypertension, and unfavorable cholesterol and glucose blood levels, which in turn may be related to a lower risk of cancer recurrence [8]. Particularly, the consumption of vegetables has been associated with a lower risk of cancer recurrence among breast cancer survivors [9]. Moreover, a growing body of evidence has shown that cancer survivors’ QoL can be improved by adopting and maintaining a healthy lifestyle [7–14]. For example, physical activity (PA) has been shown to improve psychological outcomes, fatigue, body composition, walking distance, aerobic fitness, strength, and QoL domains [15–17]. In particular, aerobic exercise with moderate-intensity appeared to be a strong positive factor affecting fatigue, walking endurance, and cancer mortality [18, 19]. According to the World Cancer Research Fund/American Institute for Cancer Research (WCRF/AICR) and the American Cancer Society [19–21], adult cancer survivors should avoid inactivity. It is recommended that cancer survivors engage in at least 150 min per week (min p/w) of moderate PA, spread throughout at least 5 days of the week, or perform 75 min p/w of vigorous PA (or an equivalent combination). A healthy diet should include at least five servings of fruit and vegetables daily [20]. Despite the beneficial effects of recommended PA and healthy diet, the great majority of cancer survivors fail to meet the lifestyle recommendations [22–25].

Meeting the individual needs of a growing number of cancer survivors, including the promotion of a healthy lifestyle, is challenging. Therefore, fully automated web-based interventions may be appropriate for providing a large population with easily accessible and low-cost support that can be personalized by applying computer tailoring [26–29]. Moreover, web-based interventions fit with the increasing demand for online health-related information, may stimulate self-care, and might complement a stepped care approach within cancer aftercare [3, 27, 30–32]. Although a growing number of web-based self-management interventions have been developed for cancer survivors in recent years, only a few web-based studies evaluated PA and/or diet outcomes [33–37]. Post intervention increases in moderate PA and/or vegetable intake have been reported, however, behavior change was not maintained at 6-month follow-up [36–39]. Although younger, female, and higher educated survivors often participated in web-based interventions, it is unknown whether and which possible subgroups of cancer survivors might benefit most from web-based lifestyle interventions [26]. This can be important knowledge to integrate web-based interventions into cancer aftercare.

The web-based intervention *Kanker Nazorg Wijzer* (Cancer Aftercare Guide, KNW) is a computer-tailored intervention that ultimately aims to increase survivors’ QoL [40]. The online portal comprises eight separate modules that target the topics PA, diet, smoking cessation, return-to-work, fatigue, anxiety and depression, social relationships, and residual problems. Survivors of various types of cancer had access to the fully automated web portal for 6 months. Previously reported findings revealed strong indications that having access to the KNW may account for meaningful increases in moderate PA and vegetable consumption after 6 months of web-access, while using the behavior specific modules accounted for higher increases of moderate PA and higher fruit and fish consumption [41].

The aim of the present study is to examine the long-term (12-month) effects of the web-based KNW on moderate PA and vegetable consumption, in order to evaluate whether the KNW outcomes on moderate PA and vegetable intake that were found 6 months after the baseline measurement were maintained in the long term. In addition, we explored whether possible effects on the behavioral outcomes (i.e. moderate PA, vegetable intake) were influenced by whether or not participants visited the module that was directed at the behavior in question. This procedure was in line with the 6-month evaluation [41]. To identify possible subgroups that might benefit most from this intervention, we explored whether a possible intervention effect was moderated by gender, age, and educational level.

## Methods

### Trial design and setting

The long-term effects of the KNW on moderate PA and vegetable consumption were assessed by conducting a two-armed randomized controlled trial including an intervention condition (IC) and a usual care waiting list control condition (UC). After centralized registration, randomization of the participants (ratio of 1:1) was automatically performed by means of a digital randomizer at the first login to the KNW [42]. Self-reported baseline assessment and the follow-up measurements with validated instruments after 3, 6, and 12 months were conducted online. In the current study, data from baseline and 6 and 12-month follow-up were included into the analyses. The IC had access to the KNW throughout 6 months, while the UC received access to the KNW after completing the 12-month measurement. Blinding participants and researchers was not possible within this eHealth trial [43]. Ethical approval for this trial (Dutch Trial Register NTR3375) was obtained from the Medical Research Ethics Committee Zuyderland-Zuyd (NL41445.096.12, 12-T-115). After approval, the board of directors of each hospital endorsed the execution of the study.

### Participants and procedure

Eligible individuals were adult (≥ 18 years of age), Dutch-speaking cancer survivors, diagnosed with various types of cancer, and who had completed primary cancer treatment (surgery, chemo- or radiation therapy) with curative intent at least 4 weeks, and up to 56 weeks prior to initial participation. Individuals with signs of cancer recurrence or severe medical, psychiatric, or cognitive disorders were excluded from participation.

Details of the recruitment procedures have been published elsewhere [41, 44]. In short, eligible cancer survivors were recruited from November 2013 through June 2014 by medical staff from 21 Dutch outpatient clinics (internal medicine, oncology, gynecology, urology, breast cancer care) during medical consultations and by reviewing patient files. A trial information package was provided, in person or by post, including comprehensive information about the trial and about scientific research [45], an informed consent form, a short log-in instruction guide, and a storage card with contact details and personal login codes to the KNW online baseline questionnaire. One reminder letter was send after 2 weeks, reminding subjects to participate in the study and to return the signed informed consent form. Data from respondents who did not return the informed consent form were excluded from analysis.

### Intervention

**Fig. 1:**
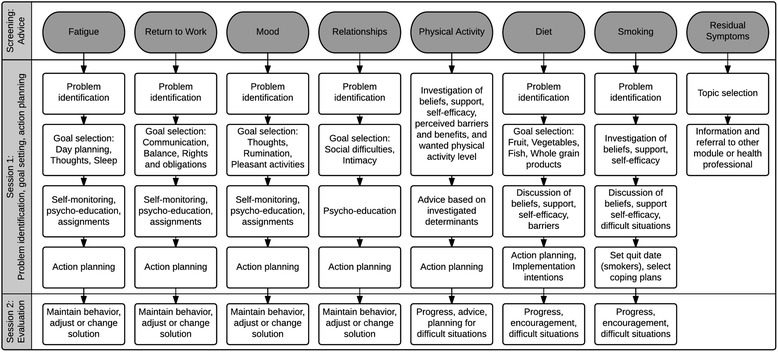
Overview of the scope and sequence of the KNW modules. From Willems et al. (2015). ©2015 Willems et al. Reprinted with permission

The KNW is a web-based self-management program that operates without human involvement. Comprehensive descriptions of the intervention and technical details are published elsewhere [40, 41]. To achieve behavior change, specific determinants and behavior change methods were applied that derived from social cognitive behavior change theories and models, such as the Theory of Planned Behavior [46], the Self-regulation Theory [47], and the Integrated Model for Change (I-Change Model) [48]. According to these theories, health behavior change is a dynamic process with a series of awareness, behavior initiation, routinizing, and maintenance phases. This process is influenced by pre-motivational determinants (e.g., knowledge, risk perception, awareness), motivational determinants (e.g., intention, attitude, self-efficacy, social influences), and post motivational determinants (e.g., ability to prepare and execute plans to achieve goals and to overcome potential barriers) [49–51]. The KNW self-management modules are PA, diet, smoking cessation, return-to-work, social relationships, fatigue, and anxiety and depression. The eighth module comprises generic information on the most common residual problems (Fig. 1). After completing the baseline assessment, the IC received feedback on their reported (lifestyle) scores by comparing them with the guidelines, including advice on what KNW modules were most relevant for them to use. This module referral advice was designed as a traffic light (red, orange, green) and was aimed at consciousness raising, an effective behavior change method to change awareness and risk perception [52]. When the PA and/or dietary guidelines were either not met or only partly met, respondents were advised to visit the corresponding module [20, 21]. Nevertheless, the respondents were free to use any module of their interest. Due to the module referral advice and the noncommittal design, it was expected that only a part of the IC participants would visit the lifestyle-modules. The module-content was personalized by means of computer tailoring and customized to personal characteristics (gender, age, marital status, children, educational level, BMI), cancer-related issues (type of cancer, type and number of comorbidities), motivational behavioral determinants (attitude, self-efficacy and intention), and current lifestyle behavior. In addition, behavior change and self-regulation methods that are relevant in maintaining behavioral changes were applied, such as providing personalized feedback, goal setting, action- and coping planning, reattribution training, and self-monitoring. All these methods were used to improve self-efficacy and to overcome possible barriers, which is in line with social cognitive behavioral change theories [47, 48, 50, 52].

Within the PA module, at first, detailed questions were asked concerning possible physical limitations, co-morbid conditions, and contraindications to vigorously intensive activity, as well as perceived barriers, social support, self-efficacy, and the pros and cons of being (more) physically active. This additional information was used to optimize the tailored feedback concerning the PA action- and coping planning. Action planning includes the when, where, and how of intended action. Coping planning refers to the mental simulation of overcoming anticipated barriers to action [51]. Participants were encouraged to gradually building up PA by setting achievable goals that fit with their capacities, to keep a record of the specified exercises, and to evaluate their activities. Videos of fellow cancer survivors and of specialized health professionals were enclosed to provide appropriate role models and information concerning different ways to be more active, how to cope with (physical) difficulties, how to overcome barriers, and how to attribute and cope with possible failures. For example, interpreting previous failures in terms of unstable attributions, and encouraging participants to resume engaging in their plan. This helps in maintaining behavior changes in the long term. The intervention mainly aimed at adopting and/or increasing moderate intensive activities (e.g. brisk walking, cycling, moderate sports activities, and household activities); however, if participants were interested, more vigorous sports activities were also encouraged, given that medical contraindications were excluded. Although respondents were encouraged to follow the PA recommendations, no specific prescriptions were provided concerning frequency, intensity, duration, and mode of specific exercises. The advice focused on sustainable behavior change by stimulating activities that fit optimally to individuals’ capabilities and preferences.

Within the diet module, additional questions explored cancer treatment-specific residual problems that might influence participants’ dietary behavior, such as changes in taste and smell, problems with chewing and swallowing, indigestion, and undesirable weight change, pain and fatigue. Moreover, the attitude toward a healthy dietary pattern (pros and cons), perceived barriers, self-efficacy, and social support concerning a more healthy diet were measured, in order to add this information to the subsequent tailoring process. Although generic information on the comprehensive diet guidelines was provided, the diet module focused on improving and maintaining healthy eating, particularly fruit, vegetable, whole grains, and fish consumption. After receiving personalized feedback on these dietary behaviors, participants could choose one or two pre-formulated goals concerning these four dietary behaviors, for example, “I want to eat sufficient vegetables (on average 200g a day)”. The personalized advice included relevant dietary information and support concerning coping with specific physical problems, possible difficult situations, and failure. Videos of fellow cancer survivors and specialized health professionals complemented the written advice, which was in line with the design of the PA module.

Four weeks after completing a module, participants were invited for a brief online, personalized evaluation session. After assessing whether participants (partly) succeeded or failed at changing the desired behavior, personalized feedback included advice on how to cope with success and failure aimed at increasing the level of coping self-efficacy in order to increase behavioral maintenance [53]. Additionally, participants were encouraged to review or adapt their action- and coping plans, in order to resume or maintain their behavior change to achieve their goals.

Besides the tailored information within the modules, valuable generic information about psychosocial and lifestyle issues was available in the form of news-items and in the user forum. Moreover, links to existing relevant websites were provided. In order to invite participants to complete questionnaires or to visit modules, several email-reminders were sent automatically with a direct link to the KNW. After the trial commencement, the intervention was applied without major adjustments, bugs, or downtimes and hyperlinks to other websites were updated when needed.

### Measurements

#### Moderate physical activity

Moderate PA was assessed using the validated self-report Short Questionnaire to Assess Health Enhancing Physical Activity (SQUASH) at baseline, after 6 months, and after 12 months [54, 55]. The 11-item SQUASH evaluates activities during commuting, leisure time, sports, household, and work. The intensity of activities was categorized into light, moderate, and vigorous. Weekly minutes of moderate PA were calculated by multiplying the number of days per week of PA with the number of minutes per day of reported moderate intensive activities. Moreover, reliability and validity of the SQUASH was confirmed in previous research among patient populations [56, 57].

#### Vegetable consumption

In the present study, vegetable consumption was measured by assessing the number of days per week (range 0-7) of vegetable consumption and the number of vegetable servings per day (one tablespoon = 50 g). These items derived from the Dutch Standard Questionnaire on Food Consumption [58] and were used at baseline, after 6 months, and after 12 months. Previous research supports the reliability and validity of a similar food frequency questionnaire assessing vegetable and fruit consumption [59]. The dependent variable, vegetable consumption in grams per day (g p/d) during 1 week (considered as an average week), was calculated by multiplying the number of days by the amount of vegetables a day (number of tablespoons × 50 grams), divided by 7 days a week.

#### Other relevant measures

Information about demographic and cancer-related characteristics was collected at baseline. Standard questions were used to measure age, gender, marital status, and education level. Employment status was dichotomized (yes/no). Type of cancer was categorized into breast, colorectal, and other types of cancer (i.e., bladder, esophageal, gynecologic, hematologic, kidney, liver, lung, prostate, stomach, testicular, and thyroid cancer). Type of treatment was categorized into surgery and chemotherapy and radiotherapy, surgery and chemotherapy, surgery and radiotherapy, and other types of treatment. Furthermore, aftercare (yes/no) and comorbidities (yes/no) were measured, and height and weight were assessed to determine body mass index (BMI). The time since completion of primary treatment, measured in weeks, was based on registry data from the hospitals. Whether participants followed the PA and diet modules was derived from program logging data. Module use was dichotomized (yes/no) and categorized into *yes* when at least the first three compulsory pages with important key information of the module were visited.

### Sample size

Since the present study is part of a larger study project, sample size calculation was based on improvements in the main outcomes and revealed that each intervention condition needed to contain 144 participants (effect size = .30; one sided α = 0.05; β = 0.2; power = .80); intra-class correlation coefficient (ICC) = 0.005). With an expected dropout of some 20–23%, the required sample size was *N* = 376 (188 per condition) at baseline.

### Statistical analysis

Baseline differences between IC and UC concerning lifestyle behaviors, and demographic and cancer-related characteristics were examined using independent t-tests and chi-square tests. Selective dropout after 12 months was assessed by applying logistic regression analysis with dropout as outcome variable (no = 0; yes = 1) and group assignment and baseline characteristics as predictive factors.

In order to evaluate the main intervention effects on moderate PA and vegetable consumption, multilevel linear regression analysis (MLA) was conducted. A three-level longitudinal data structure was used, in order to account for interdependencies. Outcomes at two time points (6 and 12-month follow-up) were clustered within the participants and participants were clustered within hospitals. Time, individuals, and hospital were added to the MLA model with a random intercept, and intervention condition and baseline value of the dependent variable were added as random slopes. The models were adjusted by adding the baseline value of the outcome behavior, standard demographic and cancer-related characteristics, significant variables from dropout analysis, and significant baseline differences. In full, the added variables were gender, age, marital status, education level, income level, employment status, BMI, type of cancer, having had cancer before, type of treatment, time since completion of primary cancer treatment, aftercare, comorbidities, vegetable intake, fruit intake, whole grain bread intake and fish intake at baseline (added as fixed intercepts). These adjustments were in line with the MLA modelling of the prior study, conducted to determine the 6-month effects of the KNW [41]. Dummy coding was used for categorical variables including more than two categories. The models were fitted by using the maximum likelihood procedure and an independent covariance structure was chosen [60]. Besides complete case analysis, intention-to-treat analysis (ITT) was also conducted [61]. Missing data of the 6-month and 12-month measurement were imputed 20 times, and multiple imputation analyses were conducted including all variables of the fully adjusted MLA models, as described above [62]. Due to the use of two outcome variables, the false discovery rate correcting procedure (FDR) was applied to account for multiple testing problems [63].

In order to evaluate whether module use, gender, age, and education possibly moderate the intervention effect, the fully adjusted 3-level MLA model was adapted as follows: To evaluate module use as a moderator, intervention condition was categorized into three categories (IC- module used, IC-module not used, control condition). To explore moderating effects of gender, age, and education, interaction terms between intervention condition and gender, intervention condition and age, and intervention condition and educational level were also added to the model. The continuous variable age was centered and dummy coding was used for educational level (three categories), with educational level *low* as reference category. The moderator analyses were conducted by using the complete cases.

Cohen’s *d* effect sizes were calculated for the main results and the results of the moderator analysis [64]. Additionally, Cohen’s *f* ^2^ was calculated in order to evaluate the local effect size within the context of the fully adjusted MLA model [64, 65]. Due to differences in the datasets of the 6-month and the 12-months follow-up (due to loss-to follow-up) and the 3-level data structure, the outcomes of the current paper varied slightly from the corresponding outcomes after 6 months [41]. All analyses were conducted using STATA version 13.1 and the calculations of the FDR corrections were conducted in R 3.2.3., base package [66].

## Results

### Study population

**Fig. 2:**
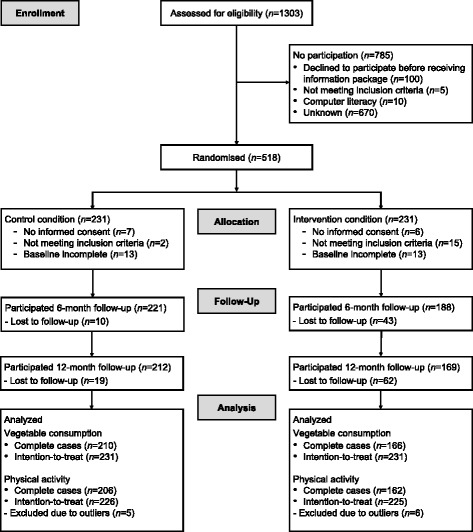
Flow diagram of participation during the study period

The flow diagram in Fig. 2 displays the study participation of the respondents during the study period. In total, 381 (82.5%) participants filled in the 12-month follow-up questionnaire and 81 (17.5%) were lost to follow-up since baseline. Dropout after 12 months was predicted by allocation to the IC (*B* = 1.805, *p* = .000), gender (male) (*B* = -1.041, *p* = .046), high income (*B* = -.866, *p* = .047), modal income (*B* = -.992, *p* = .018), ‘other’ cancer treatment (*B* = .959, *p* = .020), vigorous PA at baseline (*B* = -.001, *p* = .022), vegetable intake at baseline (*B* = -.005, *p* = .031), and fruit intake at baseline (*B* = .339, *p* = .014). MLA was corrected for all significant predictors of dropout. For the analyses of moderate PA, 11 respondents were excluded due to outliers (>6720 min p/w PA) at either baseline, 6-month or 12-month follow-up, which is in accordance with the SQUASH scorings manual, resulting in a baseline dataset of *N* = 451 for analyses [67]. Table 1 shows the demographic, cancer-related, and lifestyle-related characteristics of the participants at baseline. Mean age was 55.9 years, the majority of respondents were women (79.9%), the most represented type of cancer was breast cancer (70.6%), and more than half (53.7%) of the participants were overweight or (morbidly) obese.

**Table 1:** Baseline characteristics of the KNW study sample (*N* = 462)

	Intervention group (*N* = 231)	Control group (*N* = 231)	*P* ^1^
Demographic characteristics			
Female, *n* (%)	183 (79.2)	186 (80.5)	.728
Age, M (SD)	55.6 (11.5)	56.2 (11.3)	.596
Marital status, with partner^2^, *n* (%)	193 (83.5)	184 (79.7)	.280
Education level^3^ *n* (%)			
Low	76 (32.9)	97 (42.0)	.113
Medium	76 (32.9)	70 (30.3)	
High	79 (34.2)	64 (27.7)	
Employed, yes, *n* (%)	122 (52.8)	111 (48.1)	.306
Income below average, *n* (%)	28 (12.1)	42 (18.2)	.192
Income average, *n* (%)	84 (36.4)	78 (33.8)	
Income above average, *n* (%)	119 (51.5)	111 (48.3)	
Cancer-related characteristics			
Breast cancer, *n* (%)	162 (70.1)	164 (71.0)	.838
Other types of cancer, *n* (%)	69 (29.9)	67 (29.0)	
Treatment, *n* (%)			
Surgery, chemotherapy, radiation	86 (37.2)	108 (46.8)	.046*
Surgery, chemotherapy	61 (26.4)	48 (20.8)	
Surgery, radiation	46 (19.9)	30 (13.0)	
Other	38 (16.5)	45 (19.5)	
Aftercare, yes, *n* (%)	145 (62.8)	141 (61)	.702
Number of aftercare activities, M (SD)	1.1 (1.1)	1 (1.0)	.402
Comorbidity, yes, *n* (%)	62 (26.8)	63 (27.3)	.917
Number of comorbidities, M (SD)	0.3 (0.6)	0.4 (0.7)	.600
Time since completion primary treatment (weeks), M (SD)	25.1 (13.5)	23.4 (12.9)	.187
BMI, M (SD)	26.0 (5.0)	26.5 (4.9)	.295
BMI, *n* (%)			.593
< 18.5, underweight	2 (0.9)	3 (1.3)	
18.5–24.9, normal weight	105 (45.5)	93 (40.3)	
25.0–29.9, overweight	90 (39.0)	96 (41.6)	
30.0–34.9, obese	24 (10.4)	32 (13.9)	
≥ 40, morbidly obese	10 (4.3)	7 (3.0)	
Lifestyle-related characteristics			
Physical activity, M (SD)^4^			
Weekly days > 30 min PA;	4.9 (1.9)	4.6 (2.0)	.089
Light PA min p/w	1521.5 (897.9)	1430.2 (897.7)	.281
Moderate PA min p/w	595.9 (620.5)	526.5 (546.5)	.200
Vigorous PA min p/w	231.0 (323.9)	238.0 (426.0)	.844
Dietary behavior, M (SD)			
Vegetable intake, g p/d	138.5 (67.9)	124.2 (57.5)	.015*
Fruit intake, servings p/d	1.8 (1.2)	1.6 (1.0)	.071
Whole grain bread, slices p/d	3.1 (1.8)	2.8 (1.5)	.046*
Fish intake, servings per week	1.9 (1.9)	1.4 (1.3)	.001**

*Note*.: Abbreviations: *BMI* body mass index, *M* mean, *SD* standard deviation, *KNW* Kanker Nazorg Wijzer

^1^ *p*-value for dichotomous variables from chi-square test; for continuous variables independent *t*-test; significant result (*p* <0.05)

^2^married, cohabiting partners

^3^Low: lower vocational education, medium general secondary education; Medium: secondary vocational education, higher general secondary education; High: higher vocational education, university education

^4^Intervention group *N* = 225; Control group *N* = 226

*p < .05< **p < .01

### Main effects of the KNW on moderate physical activity after 12 months

The reported values in Table 2 are raw scores that describe the course of moderate PA at baseline, after 6 months, and after 12 months. The within group changes are displayed from baseline to 6 months (IC: M 150.7 min p/w; UC: M 72.4 min p/w) and from baseline to 12 months (IC: M 92.2 min p/w; UC: M -14.3 min p/w). After 6 months, the difference between IC and UC was 78.3 min p/w, and after 12 months, the between group difference was 106.5 min p/w. Table 3 shows the results of the MLA. The between group differences in moderate PA after 12 months were statistically significant (complete cases: *B* = 128.475, *p* = .010; ITT: *B* = 129.473, *p* = .011) with an effect size of *d* = .35; *f* ^2^ = .013. Results remained significant after correction for multiple testing.

**Table 2:** Observed means and standard deviations of moderate PA and vegetable intake per time point and group

	Baseline		6 months			12 months		
	N		N		Change from baseline	N		Change from baseline
Moderate PA		min p/w M (SD)		min p/w M (SD)	min p/w M		min p/w M (SD)	min p/w M
Intervention	225	595.9 (620.5)	178	746.6 (676.3)	150.7	162	688.1 (570.6)	92.2
Control	226	526.5 (546.5)	215	598.9 (510.7)	72.4	206	512.2 (452.1)	−14.3
Between group differences					78.3			106.5
Vegetable intake		g p/d M (SD)		g p/d M (SD)	g p/d M		g p/d M (SD)	g p/d M
Intervention	231	138.5 (67.9)	184	146.6 (56.0)	8.1	166	95.3 (44.7)	−43.2
Control	231	124.2 (57.5)	219	124.9 (60.8)	0.7	210	81.4 (44.1)	−42.8
Between group differences					7.4			0.4

*Notes*: All reported values are raw. All physical activities are moderately intensive. *PA* physical activity, *min p*/*w* minutes per week, *g*/*p*/*d* grams per day, *M* mean, *SD* standard deviation

### Main effects of the KNW on vegetable consumption after 12 months

The raw scores of the course of vegetable consumption at baseline, after 6 and 12 months are described in Table 2, as well as the within group changes after 6 months (IC: M 8.1 g p/d; UC: 0.7 g p/d), and after 12 months (IC: M -43.2 g p/d; UC: - 42.8 g p/d). The between group changes were larger after 6 months (7.4 g p/d) compared to after 12 months (0.4 g p/d). Results from MLA revealed no significant intervention effect for vegetable consumption after 12 months (*B* = 5.860, *p* = .121; ITT: *B* = 5.560, *p* = .132). This means, that the intervention effect on vegetable consumption after 6 months (*B* = 11.799, *p* = .001; ITT: *B* = 11.606, *p* = .002) was not maintained (Table 3).

**Table 3:** Main intervention effects on moderate PA and vegetable consumption after 12 months. Results of multilevel analyses

	Complete cases analysis^a^						Intention-to-treat analysis^b^			
	B	SE [95% CI]	*p*	*p.fdr*	*d* [95% CI]	*f* ^*2*^	B	SE [95% CI]	*p*	*p.fdr*
Moderate PA (min p/w)										
After 6 months	93.707	48.058 [-.48; 187.90]	.051	.051	-.25 [-.45; -.05	.008	92.886	49.233 [-3.82; 189.59]	.060	.060
After 12 months	128.475	49.627 [31.21; 225.74]	.010*	.020*	-.35 [-.55;-.14]	.013	129.473	50.393 [30.39; 228.55]	.011*	.022*
Vegetable intake (g p/d)										
After 6 months	11.799	3.667 [4.61; 18.99]	.001**	.002**	-.37 [-57; -.17]	-.013	11.606	3.781 [4.18; 19.03]	.002**	.004**
After 12 months	5.860	3.782 [-1.55; 13.27]	.121	.121	-.28 [-.49; -.08]	-.001	5.560	3.687 [-.67; 12.79]	.132	.132

Note: Multilevel analysis with three-level data structure (time, individuals, hospitals); B = Regression coefficient, *d* = Cohen’s *d, f* ^*2*^ = Cohen’s *f* ^*2*^ *. PA* physical activity; *p/w* per week, *p/d* per day. Models adjusted for gender, age, marital status, education level, income level, employment, baseline BMI, cancer type, having had cancer before, treatment type, time since last treatment, participation in aftercare, comorbidities, baseline vegetable, fruit, bread, and fish consumption

^a^ For physical activity outcomes *N* = 398; for diet outcomes *N* = 403

^b^ Imputed data: for physical activity outcomes *N* = 451; for diet outcomes *N* = 462

*p < .05; **p < .01

### Moderating effects of module use, gender, age and education

Significant effect modification was found for age (β = -9.611, SE = 3.721 [95% CI = -16.90;−2.32], *p* = .010), indicating that the KNW effect on moderate PA was significantly more likely to be higher when participants were younger. Secondary analyses with a median split (median = 57 years) confirmed this by showing that the intervention was effective among participants aged younger than 57 years after 6 months (*B* = 141.819, SE = 69.126 [95% CI = 6.34; 277.30], *p* = .040, *d* = .38, *f* ^*22*^ = .008) and after 12 months (*B* = 256.549, SE = 70.941 [95% CI = 117.51; 395.59], *p* = .000, *d* = .59, *f* ^*22*^ = .076), while it was not effective among participants aged 57 years and older (*B*_6 month_ = 38.343, SE = 65.579 [95% CI = -90.19; 166.88], *p* = .559; *B*_12 month_ = -16.047, SE = 68.337 [95% CI = -149.98; .50], *p* = .814). Among the younger participants, the increase in moderate PA at 6 months from baseline was 197.2 min p/w in the IC vs 36.1 min p/w in the UC, and after 12 months, the increase of moderate PA from baseline was 226.5 min p/w in the IC vs -49.6 in the UC (i.e. a decrease in PA). Among the older participants (≥57 years of age), the increase in moderate PA at 6 months from baseline was 102.4 min p/w in the IC vs 102.5 min p/w in the UC, and after 12 months, there was a decrease of moderate PA from baseline of -71.8 min p/w in the IC vs an increase of 33.8 min p/w in the UC. Additional tests ruled out age as a confounder.

The PA module was used by *n* = 46 (28.1%) of the IC participants who completed the 12-month follow-up questionnaire. Using the PA module did not moderate the intervention effect on the PA outcome after 6 months (β = 47.692, *SE* = 38.714 [95% CI = -28.19; 123.57], *p* = .218) and after 12 months (β = -27.736, *SE* = 39.302 [95% CI = -104.77; 49.29], *p* = .480). Furthermore, no significant moderation effects were found for gender (β = 109.065, SE = 104.309 [95% CI = -113.51; 95.38], *p* = .296) and education (β _medium_ = -8.275, SE = 94.673 [95% CI = -193.83; 177.28], *p* = .930; β _high_ = -87.163, SE = 93.508 [95% CI = -270.44; 69.11], *p* = .351).

## Discussion

The present study evaluated the 12-month effects of the web-based KNW on moderate PA and vegetable consumption. Complete cases, as well as ITT analyses, revealed that the fully automated KWN was effective in increasing and maintaining moderate PA in the long term among early cancer survivors. However, the 6-month intervention benefits on vegetable consumption did not persist in the long term.

It is very positive and promising that the effect sizes of the KNW effect on moderate PA increased over time. In contrast, Kohl et al. [26] reported in their review of reviews of Internet-delivered behavior change interventions among the general population, that 4 of 6 reviews on PA-only interventions showed that PA-only interventions were effective to increase PA. The reported effect sizes were modest and decreased during follow-up. The KNW effect on moderate PA was moderated by age. Respondents of the IC, aged younger than 57 years significantly increased moderate PA, while older participants (≥57 years) did not significantly increase moderate PA over time. The magnitude of this stratum-specific difference is of clinical importance. Higher effect sizes were found in a subgroup of participants younger than 57 years of age compared to the overall effect on moderate PA. Overall, the long-term effect of the KNW on moderate PA was clinically relevant; with an average increase of 92.2 min p/w moderate PA in the entire IC and 226.5 min p/w among younger participants. Based on this considerable PA increase and maintenance, positive long-term health benefits may be expected, such as improvements in QoL and a reduced risk of all-cause mortality [7, 12, 17, 68].

Among participants of 57 years of age and older, the intervention was not effective. This is in line with findings from Niu et al. [69], reporting that cancer survivors aged 65 years and older were less likely to meet the PA recommendations, and less likely to improve and maintain their level of PA compared with younger survivors. Age-related co-morbid conditions and physical symptoms, such as bone- or muscle loss, might impede an increase in moderate PA in older cancer survivors [70, 71]. Moreover, the web-based delivery mode and/or the content of the KNW might be more in line with relatively younger cancer survivors, who possibly better process and convert the information, with the result of higher levels of moderate PA. Possibly, cognitive skills might play a role in processing the comprehensive information supplied on the KNW portal. Recently, associations were found between age and cognitive recovery among breast cancer survivors, indicating that attention deteriorated among older survivors (≥ 60 years) [72]. This might indicate that the fully automated tailored support was well applicable for the younger subgroup. However, the older subgroup (in our analysis aged 57 years and older), might need different and/or additional support for successfully increasing moderate PA on long term.

As previously reported, there were indications that using the PA module might positively affected moderate PA outcomes after 6 months [41]. However, in the present study, no significant moderation effects for use of the PA module were found at both time points. The lack of a moderation effect of module use might be explained by the small number of PA module users, which might have affected the power of analyses. Users of the PA module were encouraged to apply behavior change methods, aiming to adopt and maintain the desired amount of PA in the long term, which may have contributed to the long-term effects [47, 48, 50]. Participants of the IC who did not use the module PA might have been triggered and encouraged to be more physically active by receiving feedback on their PA scores after completing the baseline questionnaire. Furthermore, the advantages of adopting sufficient PA in daily life were also strongly emphasized in other KNW self-management trainings modules (e.g. fatigue and mood), in the news items, and in the discussion forum.

While the KNW was beneficial in influencing vegetable consumption after 6 months, this positive effect was not maintained after 12 months. The lack of sustained long-term effect in vegetable consumption in the present study is in line with results of previously evaluated web-based interventions, which aimed to improve a healthy diet among the general population [26]. Furthermore, in populations with various chronic diseases, the majority of dietary interventions reported no statistically significant effects on dietary behavior after 12 months, even when significant short-term effects were found [73]. Bluethmann et al. [70] acknowledged that the cancer diagnosis might possibly provide a teachable moment for adopting a healthy diet, however they demonstrated that vegetable consumption continued to deteriorate as more time passes since the diagnosis. The KNW respondents started using the KNW in a period that can be considered as a teachable moment (around 6 months after completing primary cancer treatment). During the duration of the present study, the effect of the teachable moment may have become weaker, considering the improvements in fatigue and depressive symptoms among the IC within 6 months [44]. This might possibly explain the overall decrease in vegetable consumption throughout the 12-month follow-up period. In sum, the vegetable consumption of the KNW users did not sustainably improve, while a healthy dietary pattern in combination with sufficient physical activity can have a positive impact on bodyweight, comorbidities, and QoL [6, 8, 11]. To achieve sustained increases in vegetable consumption among cancer survivors, more intensive and prolonged support might be required [74–76].

The changes in moderate PA and vegetable consumption within the first 6 months of the study might be initiated by applying behavior change methods that target specific determinants that derive from social cognitive theories and behavior change models [46–48]. In their systematic review of behavior theories, Kwasnicka et al. [53] described explanations how individuals maintain behavior changes over time, in different contexts, and including the risk of a potential lapse to the prior behavior. According the authors, relevant theoretical concepts for behavior change maintenance are maintenance motives, self-regulation, psychological and physical resources, habits, and environmental and social influences. While active self-regulation is needed to initiate behavior change, conscious self-regulation may decrease and the behavior might become more habitual and effortless with repeated performance. In the present study, different cultural and environmental influences might have influenced behavior maintenance concerning moderate PA and vegetable consumption. In the Netherlands, the level of engagement in physical activity is generally high [77], while the consumption of vegetables in the general Dutch population is low an might be difficult to change [78, 79].

This fully automated KNW might be appropriate to serve as a first step in a stepped care approach within cancer aftercare. Personalized wide-ranging support can be widely disseminated at relatively low costs [30, 80]. Based on the present results on moderate PA and previously published positive outcomes of the KNW on fatigue, depression, and Qol domains, the KNW seems very promising and suitable to meet a range of aftercare needs of a large group of early cancer survivors [44]. Those cancer survivors who need more intensive treatment or additional support can be identified and referred to a subsequent program including more intensive guidance. Particularly, support is recommended to increase a healthy dietary pattern and to prevent a relapse of positive dietary changes. Moreover, based on our results, it should be taken into consideration that older cancer survivors might have different or additional support needs to increase moderate PA.

Even though current results derived from a strong study design, some limitations should be acknowledged. With regard to generalizability, it should be noted that the participants of the KNW did not represent the overall cancer survivor population. A large proportion of participants were middle-aged, female survivors of breast cancer with relative low levels of physical and psychological complaints and comorbidities, and already relatively active at start. The high number of breast cancer survivors might be due to the relative high prevalence of breast cancer, the good overall prognosis and, the well-organized breast cancer care in the Netherlands, which was helpful during the recruitment of study participants. Moreover, the intervention was directed to survivors who were able to get support from a web-based program. Prior findings confirm that web-based interventions generally reach more women than men [26]. Consequently, the results of our web-based intervention might not be generalizable to the overall cancer survivor population. Furthermore, the present outcomes might be affected by selective dropout. However, dropout was very low, especially for eHealth interventions [26]. Moreover, analyses were corrected for the corresponding variables and ITT analyses revealed comparable results to complete case analyses. Another limitation might be that the outcome variables were self-reported, allowing over- and underestimation to occur due to social desirability or recall bias [81]. Misreporting of dietary intake might also be due to misrepresentation of portion sizes and daily dietary variability [73]. As previous research suggests [82], overestimation could have occurred in moderate PA, although we used a validated measurement instrument and we accounted for over-reporting [54].

## Conclusions

Access to the web-based, fully automated KNW resulted in significant positive sustained changes of moderate PA in the long term among cancer survivors younger than 57 years of age. The increase of moderate PA was clinically relevant. Relatively older cancer survivors might, however, be in need of different or additional support in order to increase PA. Current findings that short-term increases in vegetable consumption did not sustain in the long term can be used to further improvements of the KNW. The detected low levels of vegetable intake indicate that the consumption of vegetables should be encouraged among cancer survivors, especially as more time of cancer survivorship passes. These findings add valuable information to the field, since results on long-term effects of web-based interventions on lifestyle outcomes among cancer survivors are limited. Based on these outcomes, cancer survivors can be encouraged to engage in web-based self-management interventions to increase physical activity. The KNW can complement current cancer aftercare by serving as one of the first steps in a stepped care approach.

## Abbreviations

AICR: American Institute for Cancer Research
BMI: Body mass index
FDR: False discovery rate
IC: Intervention condition
ICC: Intra-class correlation coefficient
ITT: Intention-to-treat analysis
KNW: *Kanker Nazorg Wijzer* (Cancer Aftercare Guide)
MLA: Multilevel linear regression analysis
PA: Physical activity
QoL: Quality of life
SQUASH: Short Questionnaire to Assess Health Enhancing Physical Activity
UC: Usual care waiting list control condition
WCRF: World Cancer Research Fund

## References

[1] Cancer Research UK, London. 2016. Worldwide cancer statistics. http://www.cancerresearchuk.org/health-professional/cancer-statistics/worldwide-cancer. Accessed 1 May 2016

[2] World Health Organization. 2016. Cancer Fact Sheet. http://www.who.int/mediacentre/factsheets/fs297/en/. Accessed 1 May 2016

[3] Aaronson NK, Mattioli V, Minton O, Weis J, Johansen C, Dalton SO, Verdonck-de Leeuw IM, Stein KD, Alfano CM, Mehnert A (2014). Beyond treatment - Psychosocial and behavioural issues in cancer survivorship research and practice. EJC.

[4] Deckx L, Van Abbema DL, van den Akker M, van den Broeke C, Van Driel M, Bulens P, Tjan-Heijnen VC, Kenis C, De Jonge ET, Houben B, Buntinx F (2015). A cohort study on the evolution of psychosocial problems in older patients with breast or colorectal cancer: comparison with younger cancer patients and older primary care patients without cancer. BMC Geriatr.

[5] Kenzik KM, Kent EE, Martin MY, Bhatia S, Pisu M (2016). Chronic condition clusters and functional impairment in older cancer survivors: a population-based study. J Cancer Surviv.

[6] Tsilidis KK, Papadimitriou N, Capothanassi D, Bamia C, Benetou V, Jenab M, Freisling H, Kee F, Nelen A, O’Doherty MG, et al. Burden of Cancer in a Large Consortium of Prospective Cohorts in Europe. J Natl Cancer Inst. 2016; doi:10.1093/jnci/djw127. 10.1093/jnci/djw127 27154917

[7] Schmid D, Leitzmann MF (2014). Association between physical activity and mortality among breast cancer and colorectal cancer survivors: a systematic review and meta-analysis. Ann Oncol.

[8] Bruno E, Gargano G, Villarini A, Traina A, Johansson H, Mano MP, Santucci De Magistris M, Simeoni M, Consolaro E, Mercandino A (2016). Adherence to WCRF/AICR cancer prevention recommendations and metabolic syndrome in breast cancer patients. Int J Cancer.

[9] Thomson CA, Rock CL, Thompson PA, Caan BJ, Cussler E, Flatt SW, Pierce JP (2011). Vegetable intake is associated with reduced breast cancer recurrence in tamoxifen users: a secondary analysis from the Women’s Healthy Eating and Living Study. Breast Cancer Res Treat.

[10] Husson O, Mols F, Ezendam NPM, Schep G, van de Poll-Franse LV (2015). Health-related quality of life is associated with physical activity levels among colorectal cancer survivors: a longitudinal, 3-year study of the PROFILES registry. J Cancer Surviv.

[11] Koutoukidis DA, Knobf MT, Lanceley A (2015). Obesity, diet, physical activity, and health-related quality of life in endometrial cancer survivors. Nutr Rev.

[12] Smits A, Lopes A, Das N, Bekkers R, Massuger L, Galaal K (2015). The effect of lifestyle interventions on the quality of life of gynaecological cancer survivors: A systematic review and meta-analysis. Gynecol Oncol.

[13] Carmack CL, Basen-Engquist K, Gritz ER (2011). Survivors at higher risk for adverse late outcomes due to psychosocial and behavioral risk factors. Cancer Epidemiol Biomarkers Prev.

[14] Baena Ruiz R, Salinas Hernandez P. Diet and cancer: Risk factors and epidemiological evidence. Maturitas. 2013; doi:10.1016/j.maturitas.2013.11.010. 10.1016/j.maturitas.2013.11.010 24374225

[15] Fong DY, Ho JW, Hui BP, Lee AM, Macfarlane DJ, Leung SS, Cerin E, Chan WY, Leung IP, Lam SH (2012). Physical activity for cancer survivors: meta-analysis of randomised controlled trials. BMJ.

[16] Mishra SI, Scherer RW, Snyder C, Geigle P, Gotay C (2014). Are exercise programs effective for improving health-related quality of life among cancer survivors? A systematic review and meta-analysis. Oncol Nurs Forum.

[17] Van Dijck S, Nelissen P, Verbelen H, Tjalma W, Gebruers N (2016). The effects of physical self-management on quality of life in breast cancer patients: A systematic review. Breast.

[18] Dennett AM, Peiris CL, Shields N, Prendergast LA, Taylor NF (2016). Moderate-intensity exercise reduces fatigue and improves mobility in cancer survivors: a systematic review and meta-regression. J Physiother..

[19] Inoue-Choi M, Robien K, Lazovich D (2013). Adherence to the WCRF/AICR guidelines for cancer prevention is associated with lower mortality among older female cancer survivors. Cancer Epidemiol Biomarkers Prev.

[20] Rock CL, Doyle C, Demark-Wahnefried W, Meyerhardt J, Courneya KS, Schwartz AL, Bandera EV, Hamilton KK, Grant B, McCullough M (2012). Nutrition and physical activity guidelines for cancer survivors. CA Cancer J Clin.

[21] World Cancer Research Fund/American Institute for Cancer Research. Food, Nutrition, and Physical Activity: a Global Perspective. In: Policy and Action for Cancer Prevention. Washington DC. 2009. http://www.wkof.nl/sites/default/files/Policy_Report.pdf. Accessed 1 May 2016.

[22] Kanera IM, Bolman CA, Mesters I, Willems RA, Beaulen AA, Lechner L (2016). Prevalence and correlates of healthy lifestyle behaviors among early cancer survivors. BMC Cancer.

[23] Blanchard CM, Courneya KS, Stein K, American Cancer Society’s SCS, II (2008). Cancer survivors’ adherence to lifestyle behavior recommendations and associations with health-related quality of life: results from the American Cancer Society’s SCS-II. J Clin Oncol.

[24] DeNysschen C, Brown JK, Baker M, Wilding G, Tetewsky S, Cho MH, Dodd MJ (2015). Healthy Lifestyle Behaviors of Breast Cancer Survivors. Clin Nurs Res.

[25] Mowls DS, Brame LS, Martinez SA, Beebe LA (2016). Lifestyle behaviors among US cancer survivors. J Cancer Surviv.

[26] Kohl LF, Crutzen R, De Vries NK (2013). Online prevention aimed at lifestyle behaviors: a systematic review of reviews. J Med Internet Res.

[27] Chou WY, Liu B, Post S, Hesse B (2011). Health-related Internet use among cancer survivors: data from the Health Information National Trends Survey, 2003-2008. J Cancer Surviv.

[28] Noar SM, Benac CN, Harris MS (2007). Does tailoring matter? Meta-analytic review of tailored print health behavior change interventions. Psychol Bull.

[29] de Vries H, Brug J (1999). Computer-tailored interventions motivating people to adopt health promoting behaviours: introduction to a new approach. Patient Educ Couns.

[30] Krebber AM, Leemans CR, De Bree R, Van Straten A, Smit F, Smit EF, Becker A, Eeckhout GM, Beekman AT, Cuijpers P, Verdonck-de Leeuw IM (2012). Stepped care targeting psychological distress in head and neck and lung cancer patients: a randomized clinical trial. BMC Cancer.

[31] Runowicz CD, Leach CR, Henry NL, Henry KS, Mackey HT, Cowens-Alvarado RL, Cannady RS, Pratt-Chapman ML, Edge SB, Jacobs LA (2016). American Cancer Society/American Society of Clinical Oncology Breast Cancer Survivorship Care Guideline. J Clin Oncol.

[32] Given CW, Given BA (2013). Symptom management and psychosocial outcomes following cancer. Semin Oncol.

[33] Kim AR, Park HA (2015). Web-based Self-management Support Interventions for Cancer Survivors: A Systematic Review and Meta-analyses. Stud Health Technol Inform.

[34] Kuijpers W, Groen WG, Aaronson NK, Van Harten WH (2013). A systematic review of web-based interventions for patient empowerment and physical activity in chronic diseases: relevance for cancer survivors. J Med Internet Res.

[35] Goode AD, Lawler SP, Brakenridge CL, Reeves MM, Eakin EG (2015). Telephone, print, and Web-based interventions for physical activity, diet, and weight control among cancer survivors: a systematic review. J Cancer Surviv.

[36] Bantum EO, Albright CL, White KK, Berenberg JL, Layi G, Ritter PL, Laurent D, Plant K, Lorig K (2014). Surviving and thriving with cancer using a Web-based health behavior change intervention: randomized controlled trial. J Med Internet Res.

[37] Lee MK, Yun YH, Park HA, Lee ES, Jung KH, Noh DY (2014). A Web-based self-management exercise and diet intervention for breast cancer survivors: pilot randomized controlled trial. Int J Nurs Stud.

[38] Valle CG, Tate DF, Mayer DK, Allicock M, Cai J (2013). A randomized trial of a Facebook-based physical activity intervention for young adult cancer survivors. J Cancer Surviv.

[39] Rabin C, Dunsiger S, Ness KK, Marcus BH (2011). Internet-Based Physical Activity Intervention Targeting Young Adult Cancer Survivors. J Adolesc Young Adult Oncol.

[40] Willems RA, Bolman CA, Mesters I, Kanera IM, Beaulen AA, Lechner L (2015). The Kanker Nazorg Wijzer (Cancer Aftercare Guide) protocol: the systematic development of a web-based computer tailored intervention providing psychosocial and lifestyle support for cancer survivors. BMC Cancer.

[41] Kanera IM, Bolman CA, Willems RA, Mesters I, Lechner L. Lifestyle-related effects of the web-based Kanker Nazorg Wijzer (Cancer Aftercare Guide) intervention for cancer survivors: a randomized controlled trial. J Cancer Surviv. 2016; doi:10.1007/s11764-016-0535-6. 10.1007/s11764-016-0535-6 PMC5018034 26984534

[42] OverNite Software Europe**.** TailorBuilder. 2016. http://www.ose.nl/nl/tailorbuilder.html. Accessed 1 July 2013-2016.

[43] Baker TB, Gustafson DH, Shaw B, Hawkins R, Pingree S, Roberts L, Strecher V (2010). Relevance of CONSORT reporting criteria for research on eHealth interventions. Patient Educ Couns.

[44] Willems RA, Bolman CA, Mesters I, Kanera IM, Beaulen AA, Lechner L. Short-term effectiveness of a web-based tailored intervention for cancer survivors on quality of life, anxiety, depression, and fatigue: randomized controlled trial. Psychooncology. 2016; doi:10.1002/pon.4113. 10.1002/pon.4113 26988800

[45] Medisch-wetenschappelijk onderzoek: algemene informatie voor de proefpersoon. Ministerie van Volksgezondheid. 2014. https://www.rijksoverheid.nl/documenten/brochures/2014/09/01/medisch-wetenschappelijk-onderzoek-algemene-informatie-voor-de-proefpersoon.

[46] Ajzen I (2011). The theory of planned behaviour: reactions and reflections. Psychol Health.

[47] Baumeister RF, Heatherton TF, Tice DM (1994). Losing Control: How and Why People Fail at Self-Regulation.

[48] de Vries H, Mudde A, Leijs I, Charlton A, Vartiainen E, Buijs G, Clemente MP, Storm H, Gonzalez Navarro A, Nebot M (2003). The European Smoking Prevention Framework Approach (EFSA): an example of integral prevention. Health Educ Res.

[49] Bolman C, Eggers SM, Van Osch L, Te Poel F, Candel M, De Vries H (2015). Is Action Planning Helpful for Smoking Cessation? Assessing the Effects of Action Planning in a Web-Based Computer-Tailored Intervention. Subst Use Misuse.

[50] de Vries H, Eggers SM, Bolman C (2013). The role of action planning and plan enactment for smoking cessation. BMC Public Health.

[51] Sniehotta FF, Schwarzer R, Scholz U, Schüz B (2005). Action planning and coping planning for long-term lifestyle change: theory and assessment. Eur J Soc Psychol.

[52] Kok G, Gottlieb NH, Peters GY, Mullen PD, Parcel GS, Ruiter RA, Fernandez ME, Markham C, Bartholomew LK (2015). A taxonomy of behaviour change methods: an Intervention Mapping approach. Health Psychol Rev.

[53] Kwasnicka D, Dombrowski SU, White M, Sniehotta F (2016). Theoretical explanations for maintenance of behaviour change: a systematic review of behaviour theories. Health Psychol Rev.

[54] Wendel-Vos GCW, Schuit AJ, Saris WHM, Kromhout D (2003). Reproducibility and relative validity of the short questionnaire to assess health-enhancing physical activity. J Clin Epidemiol.

[55] de Hollander EL, Zwart L, De Vries SI, Wendel-Vos W (2012). The SQUASH was a more valid tool than the OBiN for categorizing adults according to the Dutch physical activity and the combined guideline. J Clin Epidemiol.

[56] Wagenmakers R, van den Akker-Scheek I, Groothoff JW, Zijlstra W, Bulstra SK, Kootstra JW, Wendel-Vos GC, Van Raaij JJ, Stevens M (2008). Reliability and validity of the short questionnaire to assess health-enhancing physical activity (SQUASH) in patients after total hip arthroplasty. BMC Musculoskelet Disord.

[57] Arends S, Hofman M, Kamsma YP, van der Veer E, Houtman PM, Kallenberg CG, Spoorenberg A, Brouwer E (2013). Daily physical activity in ankylosing spondylitis: validity and reliability of the IPAQ and SQUASH and the relation with clinical assessments. Arthritis Res Ther.

[58] van den Brink CL, Ocké MC, Houben AW, van Nierop P, Droomers M. Validering van standaardvraagstelling voeding voor Lokale en Nationale Monitor Volksgezondheid. National Institute for Public Health and Environment. 2005. http://www.rivm.nl/Documenten_en_publicaties/Wetenschappelijk/Rapporten/2005/augustus/Validering_van_standaardvraagstelling_voeding_voor_Lokale_en_Nationale_Monitor_Volksgezondheid. Accessed 1 Oct 2013

[59] Bogers RP, Van Assema P, Kester AD, Westerterp KR, Dagnelie PC (2004). Reproducibility, validity, and responsiveness to change of a short questionnaire for measuring fruit and vegetable intake. Am J Epidemiol.

[60] Twisk J (2006). Applied Multilevel Analysis. A Practical Guide.

[61] Montori VM, Guyatt GH (2001). Intention-to-treat principle. CMAJ: Canadian Medical Association Journal.

[62] Enders CK (2010). Applied Missing Data Analysis.

[63] Benjamini Y, Yekutieli D (2001). The control of the false discovery rate in multiple testing under dependency. Ann Statist.

[64] Cohen J (1992). A power primer. Psychol Bull.

[65] Selya AS, Rose JS, Dierker LC, Hedeker D, Mermelstein RJ (2012). A Practical Guide to Calculating Cohen’s f (2), a Measure of Local Effect Size, from PROC MIXED. Front Psychol.

[66] R Development Core Team R: A Language and Environment for Statistical Computing. Vienna, Austria: the R Foundation for Statistical Computing. 2011. ISBN: 3-900051-07-0. Available online at http://www.R-project.org/. Accessed 1 June 2016

[67] Wendel-Vos W, Schuit AJ, SQUASH Short QUestionnaire to ASses Health enhancing physical activity (2004). Centrum voor Preventie en Zorgonderzoek Rijksinstituut voor Volksgezondheid en Milieu.

[68] Wen CP, Wai JP, Tsai MK, Yang YC, Cheng TY, Lee MC, Chan HT, Tsao CK, Tsai SP, Wu X (2011). Minimum amount of physical activity for reduced mortality and extended life expectancy: a prospective cohort study. Lancet.

[69] Niu C, Eng L, Qiu X, Shen X, Espin-Garcia O, Song Y, Pringle D, Mahler M, Halytskyy O, Charow R (2015). Lifestyle Behaviors in Elderly Cancer Survivors: A Comparison With Middle-Age Cancer Survivors. J Oncol Pract.

[70] Bluethmann SM, Basen-Engquist K, Vernon SW, Cox M, Gabriel KP, Stansberry SA, Carmack CL, Blalock JA, Demark-Wahnefried W:. Grasping the ‘teachable moment’: time since diagnosis, symptom burden and health behaviors in breast, colorectal and prostate cancer survivors. Psychooncology. 2015; doi:10.1002/pon.3857. 10.1002/pon.3857 PMC4698101 26060053

[71] Kampshoff CS, Stacey F, Short CE, Van Mechelen W, Chinapaw MJ, Brug J, Plotnikoff R, James EL, Buffart LM (2016). Demographic, clinical, psychosocial, and environmental correlates of objectively assessed physical activity among breast cancer survivors. Support Care Cancer.

[72] Huang Z, Zheng Y, Bao P, Cai H, Hong Z, Ding D, Jackson J, Shu XO, Dai Q. Aging, obesity, and post-therapy cognitive recovery in breast cancer survivors. Oncotarget. 2016; doi:10.18632/oncotarget.12565 10.18632/oncotarget.12565 PMC5355351 27738306

[73] Desroches S, Lapointe A, Ratte S, Gravel K, Legare F, Turcotte S (2013). Interventions to enhance adherence to dietary advice for preventing and managing chronic diseases in adults. Cochrane Database Syst Rev.

[74] Ottenbacher AJ, Day RS, Taylor WC, Sharma SV, Sloane R, Snyder DC, Lipkus IM, Jones LW, Demark-Wahnefried W (2012). Long-term physical activity outcomes of home-based lifestyle interventions among breast and prostate cancer survivors. Support Care Cancer.

[75] von Gruenigen VE, Courneya KS, Gibbons HE, Kavanagh MB, Waggoner SE, Lerner E (2008). Feasibility and effectiveness of a lifestyle intervention program in obese endometrial cancer patients: a randomized trial. Gynecol Oncol.

[76] Christy SM, Mosher CE, Sloane R, Snyder DC, Lobach DF, Demark-Wahnefried W (2011). Long-term dietary outcomes of the FRESH START intervention for breast and prostate cancer survivors. J Am Diet Assoc.

[77] Loyen A, Van Hecke L, Verloigne M, Hendriksen I, Lakerveld J, Steene-Johannessen J, Vuillemin A, Koster A, Donnelly A, Ekelund U (2016). Variation in population levels of physical activity in European adults according to cross-European studies: a systematic literature review within DEDIPAC. Int J Behav Nutr Phys Act.

[78] Volksgezondheidenzorg.info. Internationale verschillen in voeding bij volwassenen. National Institute for Public Health and Environment. 2016. https://www.volksgezondheidenzorg.info/onderwerp/voeding/regionaal-internationaal/internationale-verschillen#node-internationale-verschillen-voeding-bij-volwassenen

[79] Geurts M, Beukers M, van Rossum C. In: Consumptie groenten, fruit, vis en een aantal nutriënten opgedeeld naar opleidingsniveau en verstedelijking. National Institute for Public Health and Environment. 2013. https://www.volksgezondheidenzorg.info/onderwerp/voeding/cijfers-context/bevolkingsgroepen#node-groente-en-fruitconsumptie-naar-opleidingsniveau-volwassenen. Accessed 2 Jan 2017.

[80] Ritterband LM, Tate DF (2009). The science of internet interventions. Introduction Ann Behav Med.

[81] Van Assema P, Brug J, Ronda G, Steenhuis I, Oenema A (2002). A short dutch questionnaire to measure fruit and vegetable intake: relative validity among adults and adolescents. Nutr Health.

[82] Vassbakk-Brovold K, Kersten C, Fegran L, Mjaland O, Mjaland S, Seiler S, Berntsen S (2016). Cancer patients participating in a lifestyle intervention during chemotherapy greatly over-report their physical activity level: a validation study. BMC Sports Sci Med Rehabil.

